# Direct Visualization of Wavelength-Dependent Single Dipoles Generated on Single Gold Nanourchins with Sharp Branches

**DOI:** 10.1186/s11671-018-2675-2

**Published:** 2018-08-29

**Authors:** Geun Wan Kim, Ji Won Ha

**Affiliations:** Advanced Nano-Bio-Imaging and Spectroscopy (ANBIS) Laboratory, Department of Chemistry, University of Ulsan, 93 Daehak-Ro, Nam-Gu, Ulsan, 44610 South Korea

**Keywords:** Single particle spectroscopy, Gold nanourchins, Defocused imaging, Localized surface plasmon resonance, Dark-field microscopy

## Abstract

**Abstract:**

We present the optical properties of singe gold nanourchins (AuNUs) with sharp branches on their surfaces under dark-field (DF) microscopy and spectroscopy. The DF intensities of the single AuNUs were changed periodically as a function of the rotation angle at three localized surface plasmon resonance (LSPR) wavelengths. Furthermore, we demonstrate the generation of single dipoles with different LSPR wavelengths in multiple directions on the same AuNU surface. The multiple LSPR dipoles generated on the AuNU surface were further visualized under defocused DF microscopy and verified by characteristic doughnut-shaped defocused scattering field distributions.

**Graphical Abstract:**

ᅟ
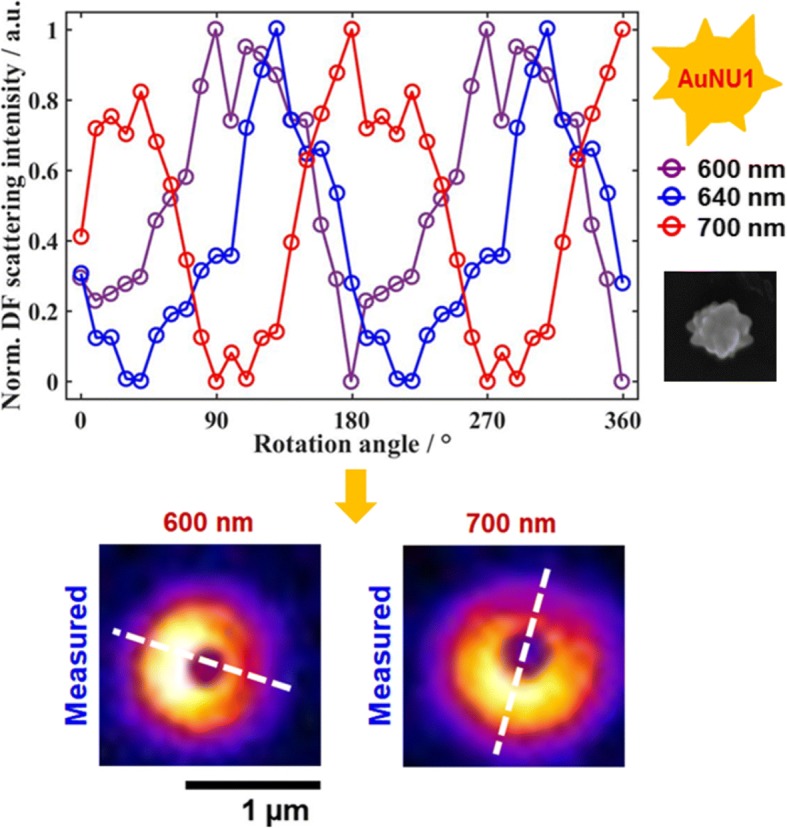

**Electronic supplementary material:**

The online version of this article (10.1186/s11671-018-2675-2) contains supplementary material, which is available to authorized users.

## Background

In recent years, plasmonic gold nanoparticles with unique optical properties have been widely employed as optical sensors [1], active surface-enhanced Raman scattering (SERS) substrates [2–7], biological and chemical sensors [8–10], and near-infrared absorbers for photothermal therapy [11, 12]. The unique size- and shape-dependent optical properties of gold nanoparticles are caused by the localized surface plasmon resonance (LSPR), which is a collective oscillation of conduction electrons on their surface with the incident light [13, 14]. Furthermore, their optical property is also dependent on the refractive index of surrounding medium [15, 16]. In particular, gold nanoparticles with multiple sharp branches are highly sensitive to changes in the dielectric constant compared to simple gold nanospheres [8, 17]. In addition, Au nanoparticles with uneven surfaces and sharp tips exhibit strong enhancement of an electromagnetic field as described by both experimental measurements and theoretical calculations [3, 18–26].

With the recent development of synthesis techniques of gold nanoparticles [27], it became possible to achieve the controlled synthesis of a range of shapes of gold nanoparticles in high yield. For example, anisotropic gold nanoparticles, such as nanourchins, nanostars [3, 8, 12, 19], nanorods [28–30], nanoplates [31], and bipyramids [32] have been synthesized and investigated on a range of applications. In particular, branched gold nanoparticles have been widely used in SERS [33, 34] and LSPR biosensors [8, 17] because of the generation of strong hot spots in the sharp branches.

To date, there have been many studies to understand the optical properties of single gold nanoparticles with sharp branches. Furthermore, theoretical calculation and experimental measurements have been used to gain a deeper insight into their size- and shape-dependent optical properties [17, 33, 35, 36]. For instance, confocal Raman imaging was employed to reveal the spatial distribution of hot spots produced in micrometer-scale single silver nanoflowers [37]. The angular distribution of a SERS imaging pattern depending on the incident wavelength has also been utilized for characterizing the particle dimers from aggregates [38]. Despite the recent studies on branched gold nanoparticles, their optical properties are not completely revealed and understood.

Very recently, to better understand the scattering properties of single gold nanourchins (AuNUs) with sharp and short branches, the polarization-dependent dark-field (DF) scattering properties of single AuNUs were investigated at their LSPR wavelength of 700 nm [39]. Single dipoles in multiple directions were found to be generated on the Au nanourchin surface at the LSPR wavelength of 700 nm. However, the effects of the incident wavelength on the change in the scattering intensity of a single AuNU have remained unanswered. In this respect, it is necessary to have a better understanding of their optical properties at the single particle level and to obtain a deeper insight into the wavelength- and polarization-dependent properties of single branched AuNUs. Furthermore, it is required to verify the generation of single dipole modes at multiple wavelengths in single AuNUs and their wavelength- and polarization-dependence on the same AuNU surface.

In the present study, we characterized the wavelength- and polarization-dependent scattering properties of single AuNUs at three different LSPR wavelengths under single-particle DF microscopy and spectroscopy. A rotational study enabled us to find polarization-dependent optical properties of the 90-nm single AuNUs at their LSPR wavelengths. Furthermore, the phase of a DF intensity trace was changed according to the incident wavelengths, which supports the generation of single dipoles with multiple LSPR wavelengths on the surface of a single AuNU with sharp tips. The generation of multiple LSPR dipoles was further visualized and verified by defocused scattering image patterns of single AuNU at the LSPR wavelengths.

## Methods/Experimental

### Materials and Sample Preparation

AuNUs with an average diameter of 90 nm were obtained from Sigma-Aldrich (St. Louis, MO, USA). The AuNU colloid solution was first diluted with 18.2-MΩ pure water to the appropriate concentration. The diluted solution was then sonicated for 15 min at room temperature. The samples were prepared by spin-casting an AuNU solution onto a pre-cleaned glass slide. Subsequently, a 22 mm × 22 mm no. 1.5 coverslip (Corning, NY) was placed on the glass slide. In this study, the concentration of AuNUs on the glass surface was controlled to approximately 1 μm^− 2^ to facilitate single-particle characterization and to minimize interparticle LSPR coupling, which can result in a spectral shift.

### Characterization

Structural characterizations of citrate-stabilized AuURs were carried out using a transmission electron microscope (TEM) (H-8100, Hitachi, Japan) and a scanning electron microscope (SEM) (JSM6500F, JEOL, Japan). Furthermore, the heterogeneous LSPR ensemble absorption spectrum of AuNUs dispersed in water was recorded with a Varian Carry 300 UV-Vis spectrometer.

### Scattering-Based Dark-Field Microscopy

DF microscopy imaging was performed under a Nikon inverted microscope (ECLIPSE Ti–U). In DF mode, the microscope utilized a Nikon Plan Fluor 100 × 0.5–1.3 oil iris objective and a Nikon DF condenser. An Andor iXonEM+ CCD camera (iXon Ultra 897) was used to record the DF images of the AuNUs. In this study, wavelength- and polarization-dependent DF scattering imaging of single AuNUs was carried out by linearly polarizing the excitation beam and changing the polarization in 10° increments. A polarizer was placed in the beam path to measure the polarization dependence of scattered light. The wavelength-dependent single particle DF studies were carried out by using the bandpass filters with a central range of 600 nm (full width at half-maximum, ± 14 nm), 640 nm (full width at half-maximum, ± 14 nm), and 700 nm (full width at half-maximum, ± 13 nm). The bandpass filters were obtained from Thorlabs (Newton, NJ) and inserted into the beam path of the microscope to illuminate the samples. A rotational study was carried out for single AuNUs at three excitation wavelengths by rotating the rotational stage at 10 ° intervals. As the stage was rotated, the fixed AuNUs were positioned at different orientations. An Andor iXonEM+ CCD camera (iXon Ultra 897) was used to record highly detailed DF scattering images of AuNUs. The collected images were analyzed using ImageJ.

### Single Particle Scattering Spectroscopy

DF scattering spectra were acquired with an Andor spectrophotometer (SHAMROCK 303i, SR-303I-A) connected with an Andor CCD camera (Newton DU920P-OE). When obtaining a spectrum, the scanning stage moved the sample to the desired location so that only scattered light from the selected location was collected by the objective. The scattered light was directed to the entrance of the spectrophotometer, dispersed by a grating (300 l/mm), and detected by the Newton CCD camera. The background was measured at a region without any particles. Data analysis was performed with specially designed MATLAB programs.

## Results and Discussion

**Fig. 1:**
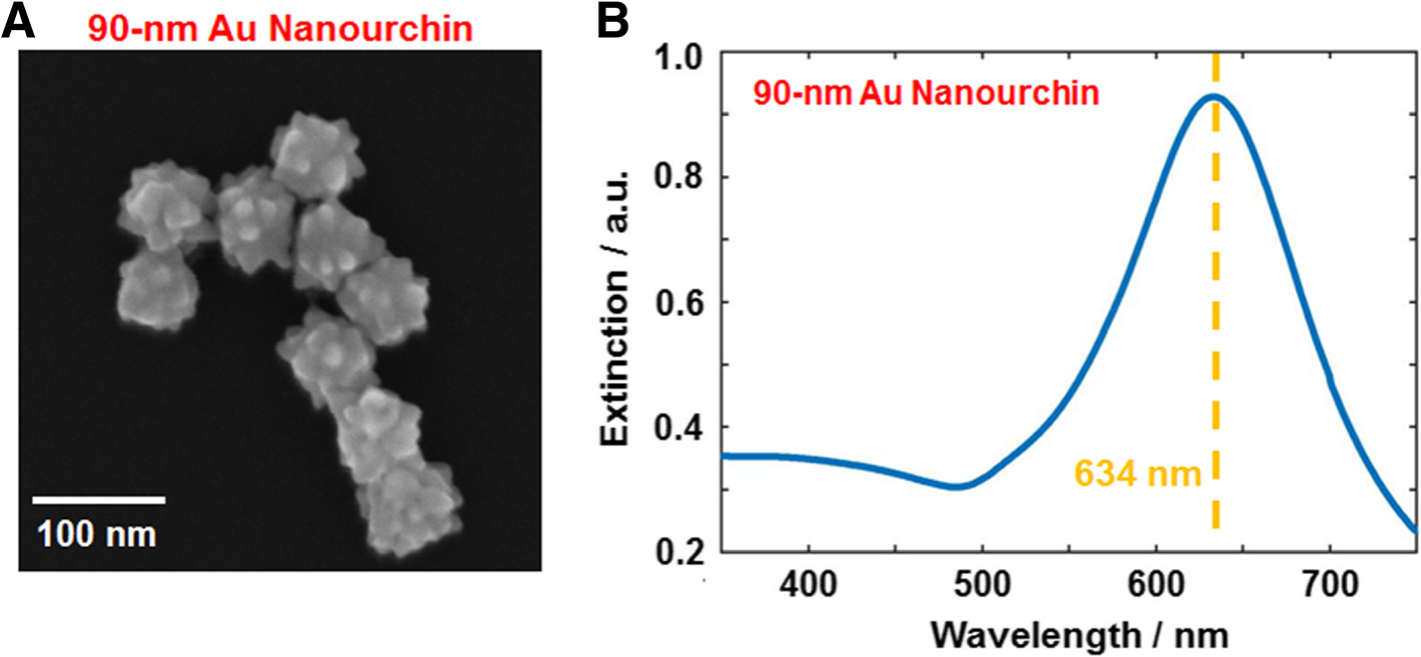
**a** SEM image of 90 nm AuNUs with sharp and short branches. **b** UV-Vis absorption spectrum of 90 nm AuNUs dispersed in water. A single broad peak is observed at approximately 634 nm indicated by the yellow-dotted line

The structures of the 90 nm AuNUs were initially characterized by scanning electron microscopy (SEM). The AuNUs have a diameter of 90 nm with short branches. The three-dimensional (3D) structure of the AuNUs with sharp branches was observed clearly in the SEM image (Fig. 1a). More SEM images are provided in Additional file 1: Figure S1 and S2. As shown in Fig. 1a and Additional file 1: Figure S1, the AuNUs have uneven and spiky surfaces because of the multiple short and sharp branches on their surface. The UV-Vis absorption spectra of AuNUs dispersed in water was then obtained (Fig. 1b). As shown in Fig. 1b, the 90 nm AuNUs showed a single broad LSPR peak at approximately 634 nm.

**Fig. 2:**
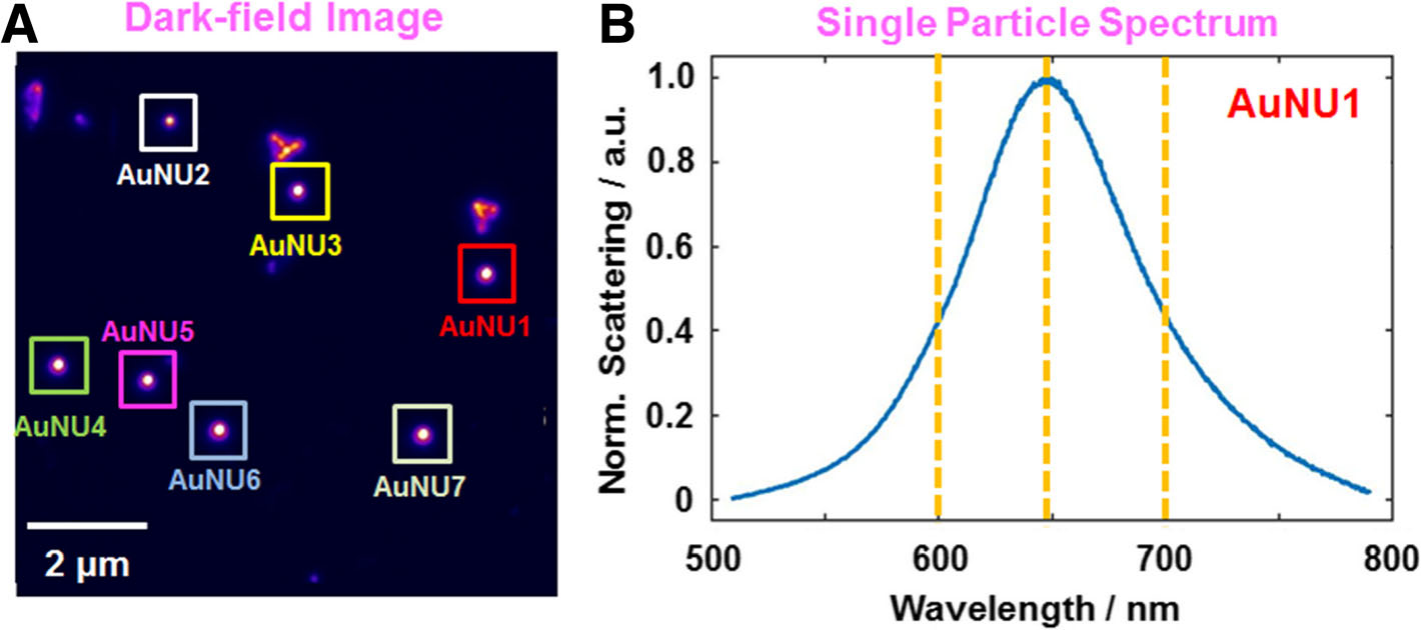
**a** DF scattering image of 90 nm AuNUs deposited on a glass slide. **b** Single particle scattering spectrum of AuNU1 highlighted with a red square in **a**. A single LSPR peak was observed around 650 nm for the AuNU1. The yellow-dotted lines indicate the incident LSPR wavelengths (600 nm, 640 nm, 700 nm) used in this study

To better understand the scattering properties of AuNUs, we performed single-particle measurements under DF microscopy and spectroscopy, which allowed us to eliminate the ensemble averaging. Additional file 1: Figure S3 shows an experimental setup for single particle microscopy and spectroscopy used in this study. A sample was prepared by spin casting a solution containing AuNUs on a pre-cleaned glass slide. In this study, we used a glass slide as a substrate because the optical properties of AuNUs are strongly dependent on the dielectric constant of substrate. The AuNUs were then measured by illuminating them with randomly polarized white light focused tightly by a high-numerical aperture (NA) oil condenser under DF microscopy (Additional file 1: Figure S4). Figure 2a shows a DF scattering image of the 90 nm AuNUs deposited on a glass slide. The single-particle scattering spectra of the AuNUs were obtained, as highlighted by squares in Fig. 2a. Figure 2b shows a single particle total scattering spectrum of AuNU1 in Fig. 2a. A single LSPR peak was observed at ~ 650 nm for AuNU1, which is consistent with the results obtained by the ensemble experiment in Fig. 1b.

In a recent study, the polarization-dependent DF scattering intensities of single AuNUs were observed at the LSPR wavelength of 700 nm [39]. Furthermore, the single AuNUs with sharp and short branches could generate single dipoles in multiple directions on the nanourchin surface at 700 nm. On the other hand, the wavelength-dependent DF scattering intensities of single AuNUs were not investigated and largely unanswered. Therefore, in this paper, we made efforts to gain deeper insight into the wavelength- and polarization-dependent scattering properties of single AuNUs with spiky and uneven surfaces. As shown in the yellow-dotted lines in Fig. 2b, three different LSPR incident wavelengths of 600 nm, 640 nm, and 700 nm were chosen and used to measure single AuNUs.

**Fig. 3:**
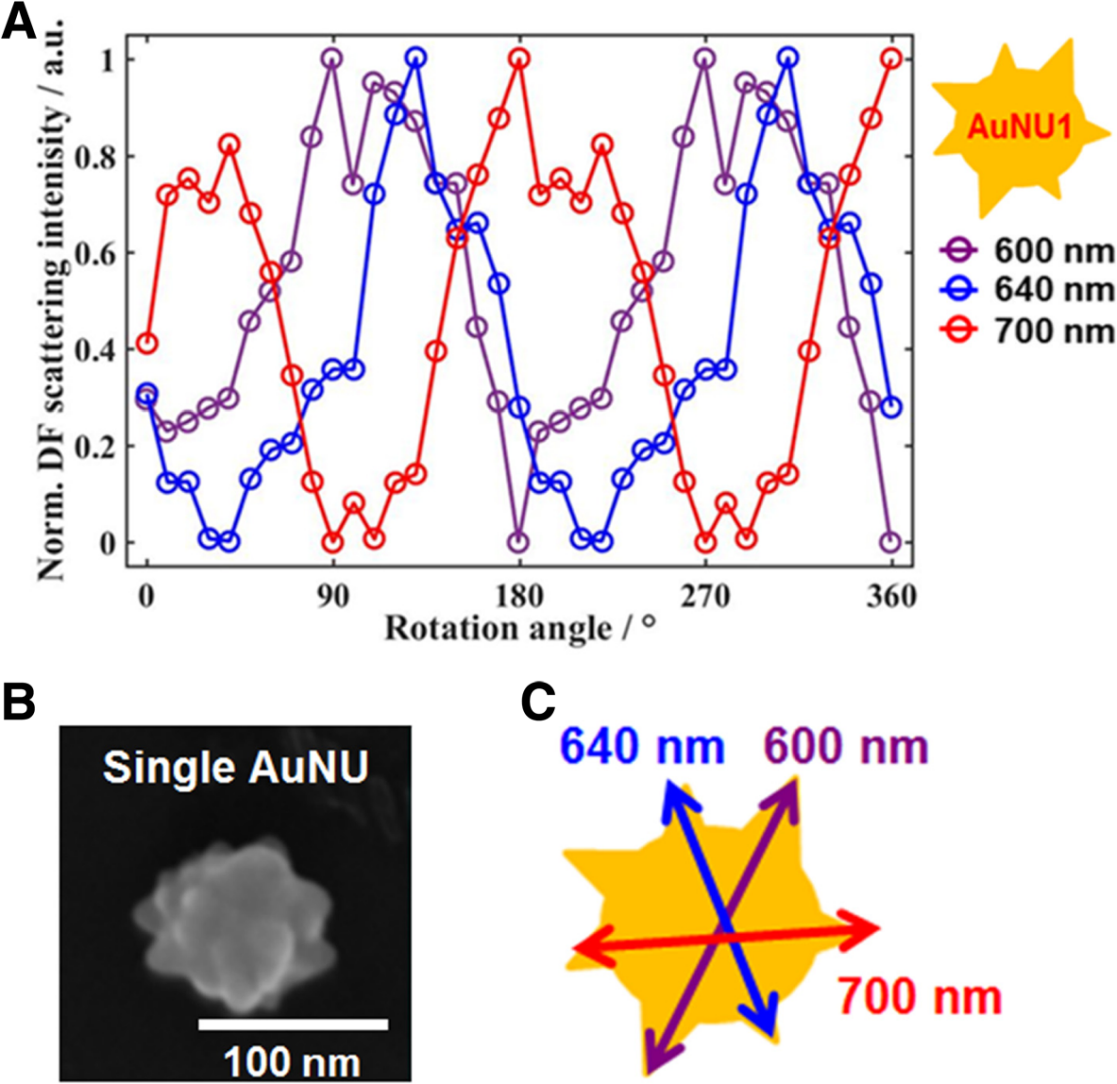
**a** Normalized DF intensities for AuNU1 at the three different LSPR wavelengths as a function of the rotational angle. **b** Enlarged SEM image of a single AuNU with a spiky uneven surface. **c** Schematic diagram showing the generation of single dipoles with different wavelengths in multiple directions on the same AuNU surface

A rotational study was carried out for single 90 nm AuNUs at the three excitation wavelengths by rotating a polarizer at intervals of 10 °. A polarizer and three bandpass filters were placed on the path of light to elucidate the polarization- and wavelength-dependent scattering properties of single AuNUs. Figure 3a presents the changes in the normalized scattering intensities of AuNU1 at the three different wavelengths (600 nm, 640 nm, and 700 nm) as a function of the rotational angle. The periodically changed DF scattering intensity was found according to the rotation angle for the three different LSPR wavelengths. On the other hand, slight fluctuations (up and down) of the DF intensities was observed, which clearly differs from the periodic changes in the DF intensities of a single gold nanorod (AuNR), behaving as a single dipole. These fluctuations can be explained by the spatial distributions of sharp and short branches.

In addition to the polarization dependence, it should be noted that AuNU1 had a different phase of DF scattering intensities depending on the excitation LSPR wavelengths, as shown in Fig. 3a. More experimental result for single AuNU6 is provided in Additional file 1: Figure S5. The results support the generation of single dipoles with different LSPR wavelengths produced in multiple directions on the same AuNU1 with short branches (Fig. 3b, c). To further support the generation of single dipoles in multiple directions on the AuNU surface, we attempted to measure single AuNUs under defocused DF scattering microscopy. Defocused orientation and position imaging (DOPI) technique is a direct and simple method with the capability of visualizing and determining three-dimensional (3D) dipole orientation of anisotropic single gold nanorods (AuNRs) [40–42]. The core idea is that the direct detection of the spatial distribution of the scattered or emitted field of single dipoles becomes possible when the imaging system is defocused deliberately by ~ 1 μm. In this study, the polar angle *θ* and the azimuthal angle *φ* of a dipole generated on the AuNU surface in 3D space are defined, as shown in Additional file 1: Figure S6A (please see the supplementary information for more details in the DOPI technique). Therefore, it is possible to resolve the 3D orientation of single dipoles generated on the AuNU surface by characterizing the characteristic scattering intensity distributions.

**Fig. 4:**
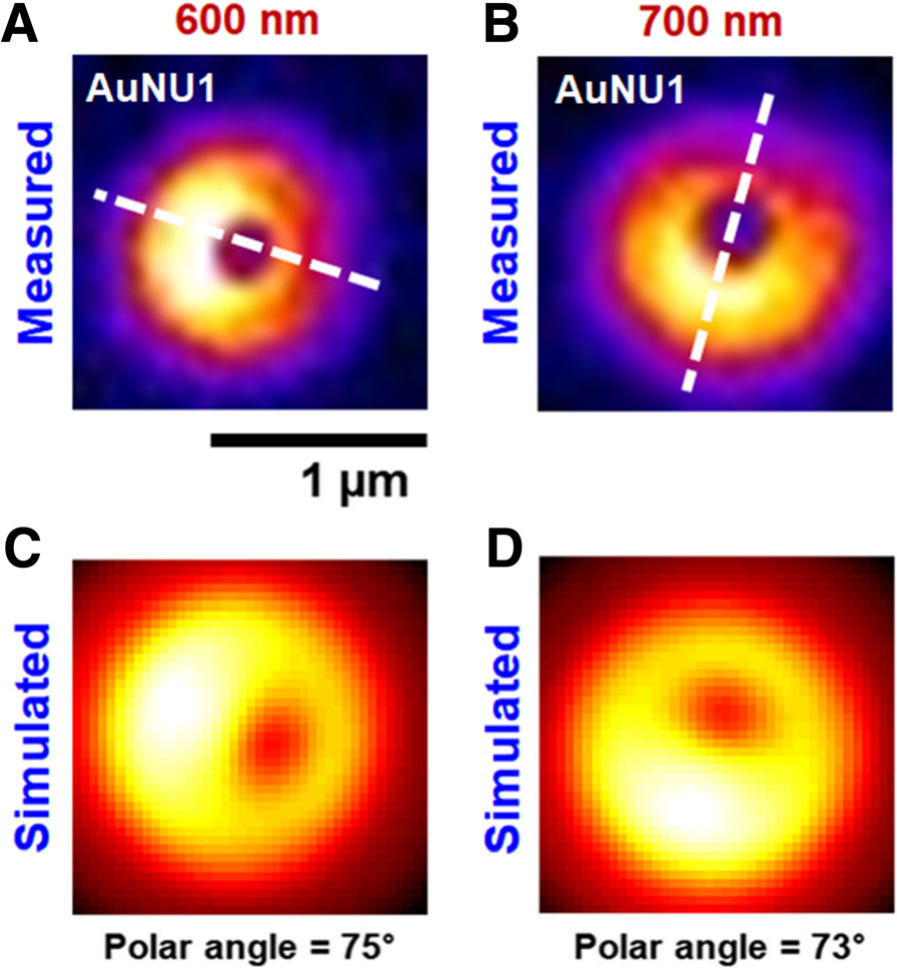
**a**, **c** Measured and best-matched simulation patterns of single AuNU1 on a glass slide at 600 nm. **b**, **d** Measured and best-matched simulation patterns of single AuNU1 at 700 nm. The white-dotted line shows the single dipole orientation generated on the AuNU surface at the LSPR wavelength. The scale bar represents 1 μm

We therefore tested if the spatial scattering field distribution of single AuNUs can be resolved directly from their defocused scattering image patterns. The AuNUs were measured at their LSPR wavelengths under defocused DF scattering microscopy. When the AuNU1 was measured at the LSPR wavelengths (600 nm and 700 nm) and positioned at ~ 1 μm away from the focal plane, characteristic doughnut-shaped scattering patterns were observed with two lobes in the peripheral area (Fig. 4a, b). Furthermore, the spatial intensity distribution on the CCD camera was no longer circularly symmetrical because single dipoles generated on the AuNU surface were tilted with the respect to a glass surface. We then tried to obtain detailed information on the 3D orientation of single dipoles generated on the surface of AuNU1 (Fig. 4a, b). The in-plane orientation angle *φ* can be extracted readily from the lobe scattering pattern exhibiting angular anisotropy as seen in the white-dotted line in Fig. 4a, b), which is consistent with the result to show the phase difference of ~ 90° in Fig. 3a. The out-of-plane polar angle *θ* was estimated using the program developed by Enderlein and Böhmer for simulating the characteristic intensity distribution from an emitter with three perpendicular emission dipoles of different emission strengths (Additional file 1: Figure S6B, see the supplementary information for more details). The 3D orientation of single dipoles on a glass substrate can be estimated by referring to their corresponding field map and the best-fit simulated scattering pattern. Figure 4c, d shows the best-fit simulated patterns at the two LSPR wavelengths of 600 nm and 700 nm. The polar angles of the generated dipoles on the AuNU1 at 600 nm and 700 nm were estimated to be about 75 ° and 73 °, respectively. Therefore, we successfully visualized the single dipoles generated in multiple directions with different LSPR wavelengths on the same AuNU surface under defocused DF microscopy.

**Fig. 5:**
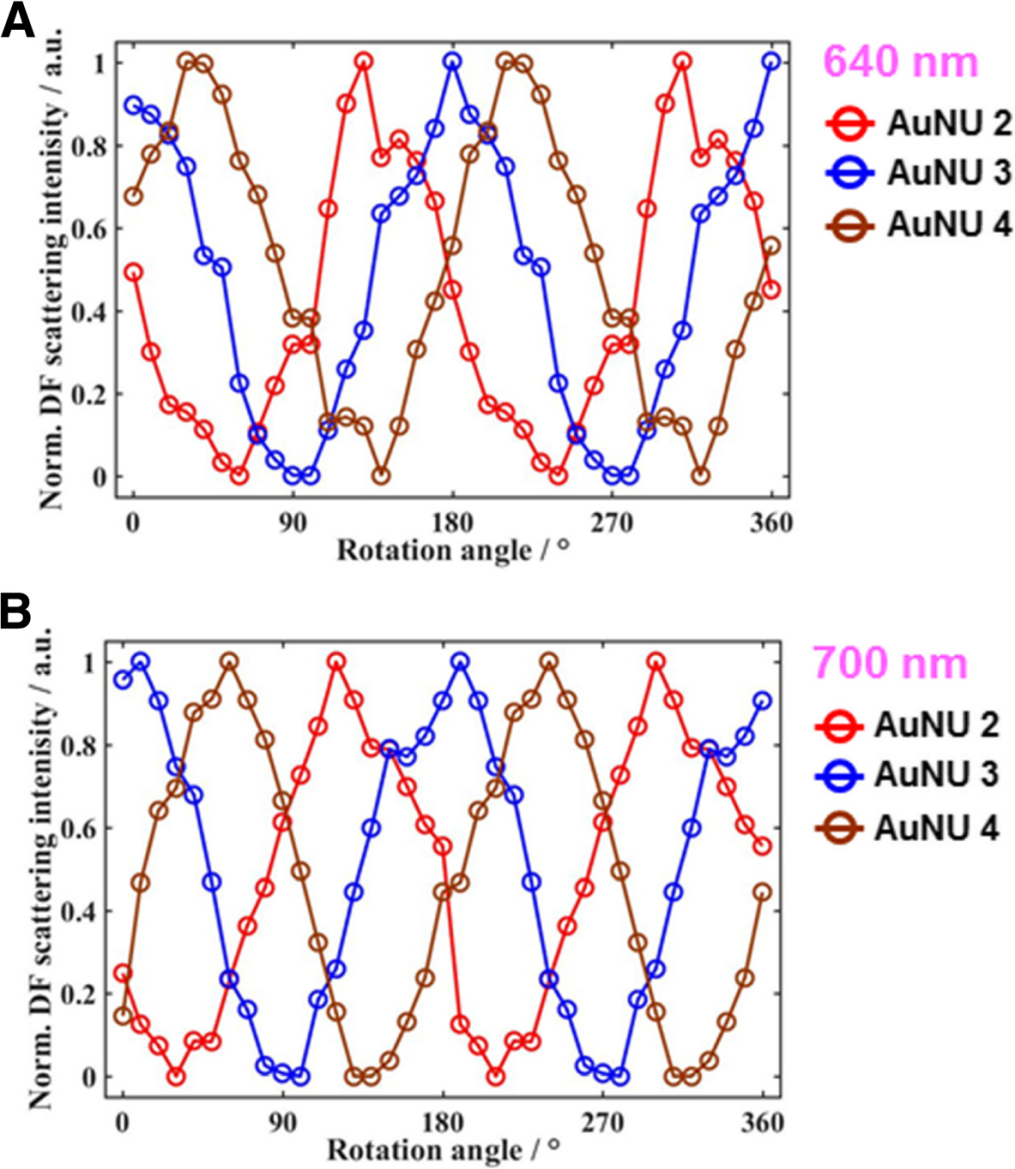
**a** Changes in the normalized scattering intensities for three AuNUs (AuNU2 to AuNU4) at 640 nm as a function of the rotational angle. **b** Changes in the normalized scattering intensities for three AuNUs (AuNU2 to AuNU4) at 700 nm as a function of the rotational angle

Last, the DF scattering intensities of single AuNUs measured at the same excitation wavelength were then compared, as shown in Fig. 2a. Figure 5a shows the DF scattering intensities of three AuNUs (AuNU2 to AuNU4) at an excitation wavelength of 640 nm. Further experimental data for other AuNUs (AuNU5 to AuNU7) at a LSPR wavelength of 640 nm are provided in Additional file 1: Figure S7. Each AuNU has a different phase as a function of the rotational angle, which indicates a different dipole orientation at 640 nm on the particle surface for the three AuNUs (AuNU2 to AuNU4). The experimental results were observed at the LSPR wavelength of 700 nm for the same AuNUs (Fig. 5b and Additional file 1: Figure S8). Therefore, this paper provides a deeper understanding of the wavelength- and polarization-sensitive optical properties of short and sharp branches on the AuNU surface under focused and defocused DF microscopy at the single particle level.

## Conclusions

The optical properties of single AuNUs with sharp and short tips on their surfaces were examined by DF microscopy and spectroscopy. The DF intensities of single AuNUs were investigated with linearly polarized light at three different LSPR wavelengths, 600 nm, 640 nm, and 700 nm, as a function of the rotation angle. The DF intensities were changed as a function of the rotational angle at three different wavelengths. More interestingly, the phase of a DF intensity trace differed according to the incident wavelengths, which can be attributed to the generation of single dipoles with different LSPR wavelengths in multiple directions on the same AuNU surface. Furthermore, we directly visualized single dipoles generated in multiple directions at the different incident wavelengths on the same AuNU surface under defocused DF microscopy. Therefore, this paper offers a deeper insight into the scattering properties of highly branched AuNUs under focused and defocused DF microscopy at the single particle level. Moreover, the knowledge gained from this study will be beneficial for various uses of branched AuNUs in SERS, LSPR biosensors, optical imaging probes, etc.

## Additional file

Additional file 1: **Figure S1.** SEM image of 90-nm AuNUs. **Figure S2.** Enlarged SEM images of 90-nm AuNUs. **Figure S3.** Photograph to show an experimental setup for single particle microscopy and spectroscopy. **Figure S4.** The working principle of dark-field (DF) microscopy and spectroscopy. **Figure S5.** Normalized DF intensities for AuNU6 at the three different LSPR wavelengths, 600 nm, 640 nm, and 700 nm, as a function of the rotational angle. **Figure S6.** (A) Schematic diagram to show the definitions of the polar angle *θ* and azimuthal angle *φ* of single dipole generated on the AuNU surface in 3D space. (B) Schematic diagram depicting three-perpendicular dipoles along the three axes. *E*_a_ denotes the scattering electric field of the nanorod along the main long axis. **Figure S7.** Normalized DF intensities for AuNUs (AuNU5 to AuNU7) at 640 nm as a function of the rotational angle. **Figure S8.** Normalized DF intensities for AuNUs (AuNU5 to AuNU7) at 700 nm as a function of the rotational angle. (DOC 2360 kb)

## Abbreviations

AuNR: Gold nanorod
AuNU: Gold nanourchin
DF: Dark-field
DOPI: Defocused orientation and position imaging
LSPR: Localized surface plasmon resonance
NA: Numerical aperture
SEM: Scanning electron microscope
TEM: Transmission electron microscope
