# Supplementary material for: Direct Visualization of Wavelength-Dependent Single Dipoles Generated on Single Gold Nanourchins with Sharp Branches
Source: Nanoscale Res Lett. 2018 Aug 29;13:256. doi: 10.1186/s11671-018-2675-2 (PMC6115324; doi:10.1186/s11671-018-2675-2)
Supplement: Supplementary file 1 — Figure S1. SEM image of 90-nm AuNUs. Figure S2. Enlarged SEM images of 90-nm AuNUs. Figure S3. Photograph to show an experimental setup for single particle microscopy and spectroscopy. Figure S4. The working principle of dark-field (DF) microscopy and spectroscopy. Figure S5. Normalized DF intensities for AuNU6 at the three different LSPR wavelengths, 600 nm, 640 nm, and 700 nm, as a function of the rotational angle. Figure S6. (A) Schematic diagram to show the definitions of the polar angle θ and azimuthal angle φ of single dipole generated on the AuNU surface in 3D space. (B) Schematic diagram depicting three-perpendicular dipoles along the three axes. Ea denotes the scattering electric field of the nanorod along the main long axis. Figure S7. Normalized DF intensities for AuNUs (AuNU5 to AuNU7) at 640 nm as a function of the rotational angle. Figure S8. Normalized DF intensities for AuNUs (AuNU5 to AuNU7) at 700 nm as a function of the rotational angle. (DOC 2360 kb) [file 11671_2018_2675_MOESM1_ESM.doc]

**Suppplementary Information**

**Direct Visualization of Wavelength-Dependent Single Dipoles Generated on Single Gold Nanourchins with Sharp Branches**

Geun Wan Kim^1^ and Ji Won Ha^1*^

^1^Advanced Nano-Bio-Imaging and Spectroscopy (ANBIS) Laboratory, Department of Chemistry, University of Ulsan, 93 Daehak-Ro, Nam-Gu, Ulsan 44610, South Korea

^*^To whom correspondence should be addressed.

**J. W. Ha**

Phone: +82-52-712-8012

Fax: +82-52-712-8002

E-mail: jwha77@ulsan.ac.kr

This document contains additional experimental methods and supplementary figures (Fig. S1 to S8).

**Experimental Section**

1. **Defocused Orientation and Position Imaging Technique.**

Anisotropic gold nanorods (AuNRs) that are much smaller than the wavelength of incident light can be considered electric point dipoles. According to an electrostatic approximation, plasmon oscillations from anisotropic AuNR can be simplified as three-perpendicular independent dipoles along the three axes (Fig. S6B). Oscillation along the long principal axis (a-axis) is defined as a longitudinal mode and the other perpendicular oscillations are defined as transverse modes vibrating along the short axes (b and c axes). *E*_a_ indicates the scattering electric field of the nanorod along the main long axis, while *E*_b_ and *E*_c_ are the scattering electric fields along the short transverse axes, b and c. The overall scattering electrical field from a AuNR can be quantified through a linear superposition of three independent scattering electric fields associated with three mutually orthogonal dipoles, as shown in Eq. (1):

$$E_{(scat)}=\sum_{a,b,c} E_{(scat)j}=E_{(scat)a}+E_{(scat)b}+E_{(scat)c}$$

However, *E*_a_ the scattering electric field of the nanorod along the main long axis (or the longitudinal dipole) is much more dominant than the two transverse dipoles. Therefore, a AuNR behaves as a single dipole character.

Defocused orientation and position imaging (DOPI) technique is a direct and simple method with the capability of visualizing and determining three-dimensional (3D) dipole orientation of anisotropic single AuNRs. The core idea is that the direct detection of the spatial distribution of the scattered or emitted field of single dipoles becomes possible when the imaging system is defocused deliberately by ~1 μm.

**2. Simulation of Scattering Image Patterns of AuNRs**

We used the simulation program developed by Enderlein and Böhmer.[^1^](#_ENREF_1) The program is designed to calculate the characteristic intensity distribution from an emitter with three perpendicular emission dipoles of different emission strength. It has been widely used to determine the spatial orientation of single dye molecules. The simulation program is a special Matlab based utility with a graphics user interface (GUI) for easy calculation. This program allows us to calculate exactly the defocused (or focused) images of single molecules. For using the GUI, one should download the files from the website (http://www.joerg-enderlein.de/imagingOfSingleMolecules.html).

The parameters that can be input are: the numerical aperture of the objective lens, magnification of imaging, extent of defocusing (or defocusing distance in micrometers), *κ* and *R*. Therefore, in this simulation, we adjusted the numerical aperture of the objective lens and magnification of imaging and defocusing. Furthermore, we adjusted the parameter *κ* and R into the program to define the emission strength ratios of the three independent dipoles (Fig. S6B). The ratio *κ* defines the ratio of the emission strength of the b- to the c-dipole (transverse dipoles, Fig. S6B) as shown below.

$$I_{b}/I_{c}=(1-\kappa)/(1+\kappa)$$

In addition, the ratio *R* defines the emission strength of the a-dipole (or longitudinal dipole) to the combined b and c dipoles (or transverse dipoles) as shown below.

$$R\times I_{a}+(1-R)\times(I_{b}+I_{c})$$

When *R* is 1, we only have the contribution from a-dipole (longitudinal dipole) to the image patterns. In the present study, we used a *R* value of 1 to simulate the scattering patterns of single dipoles generated on the AuNU surface at different LSPR excitation wavelengths.

**References**

1. Böhmer, M.; Enderlein, J. *J. Opt. Soc. Am. B* **2003,** 20, (3), 554-559.

2. Lieb, M. A.; Zavislan, J. M.; Novotny, L. *J. Opt. Soc. Am. B* **2004,** 21, (6), 1210-1215.

**Supporting Figures**

**
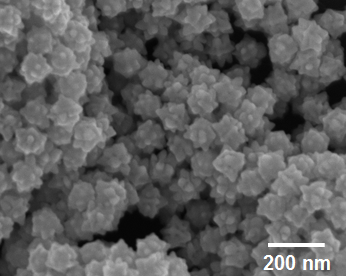
**

**Fig. S1** SEM image of 90-nm AuNUs


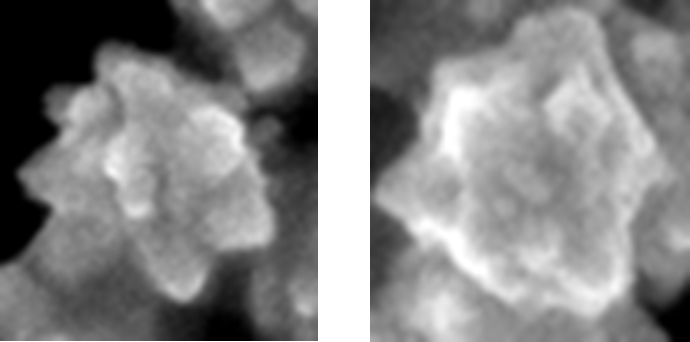


**Fig. S2** Enlarged SEM images of 90-nm AuNUs


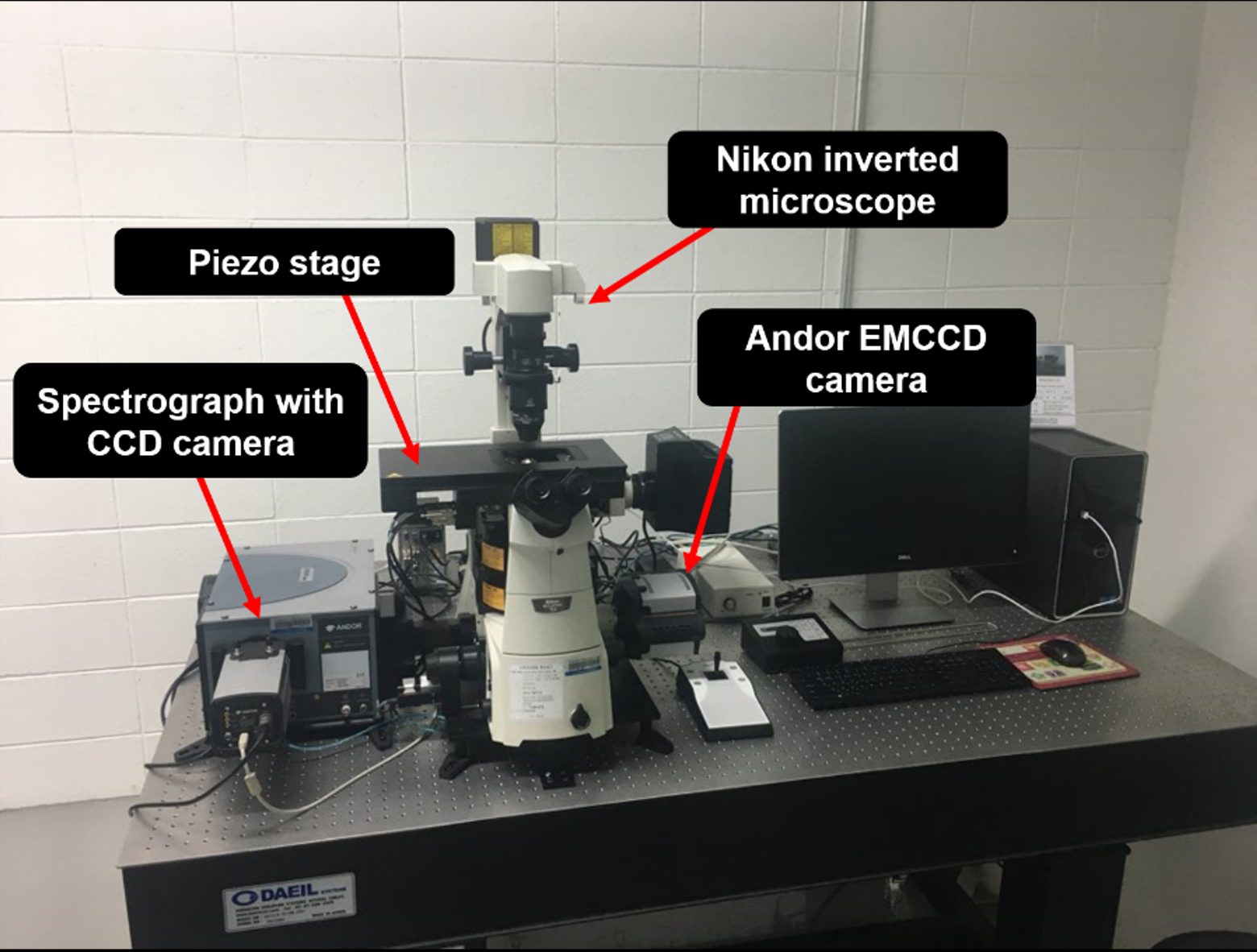


**Fig. S3** Photograph to show an experimental setup for single particle microscopy and spectroscopy


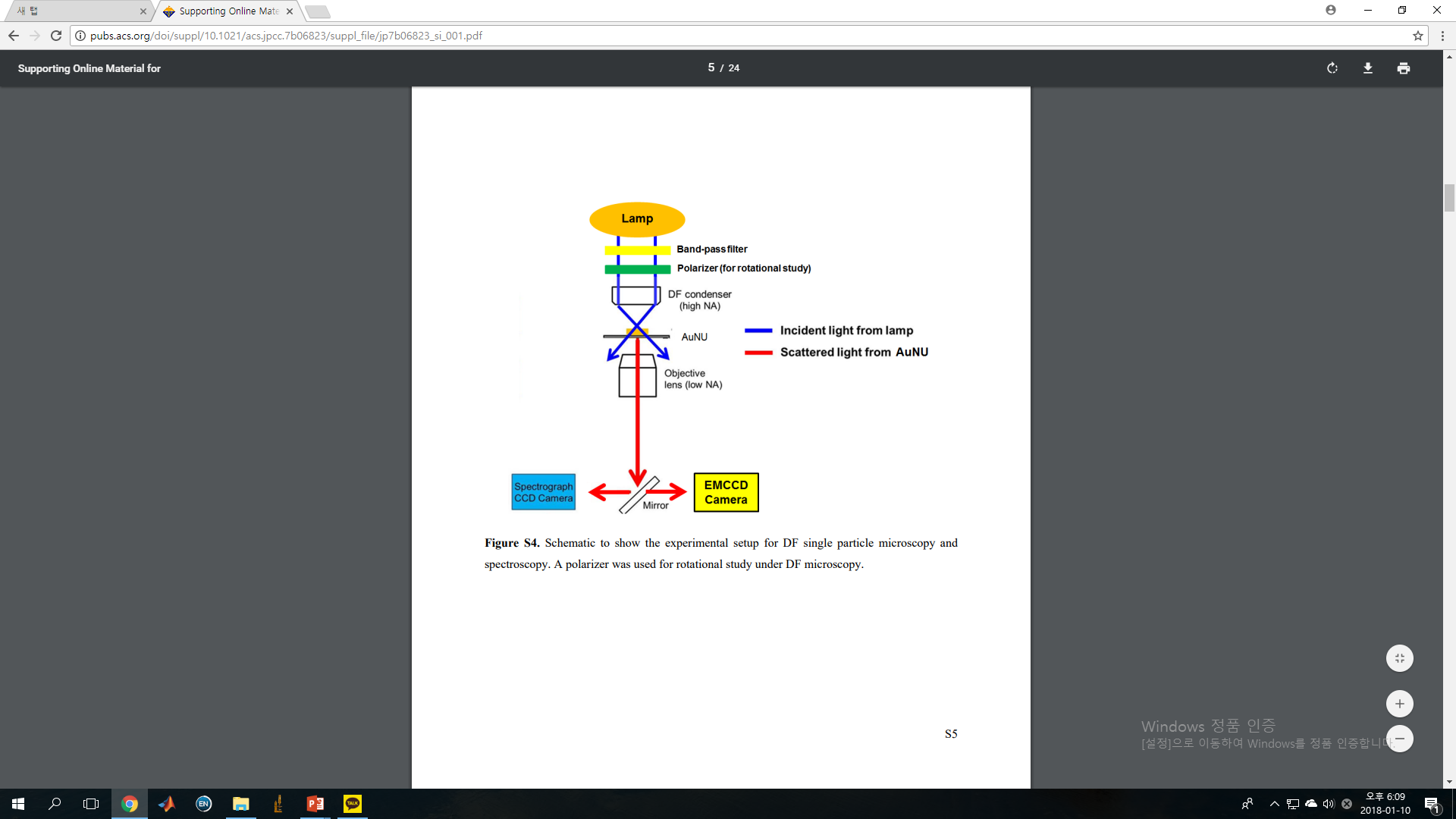


**Fig. S4** The working principle of dark-field (DF) microscopy and spectroscopy


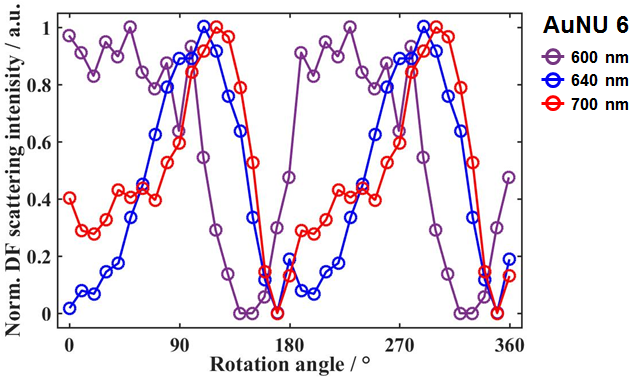


**Fig. S5** Normalized DF intensities for AuNU6 at the three different LSPR wavelengths, 600 nm, 640 nm, and 700 nm, as a function of the rotational angle


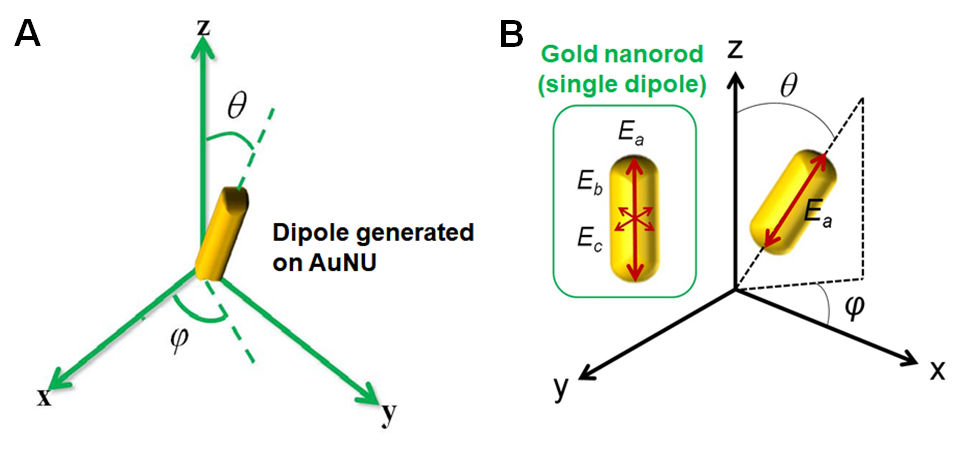


**Fig. S6** (A) Schematic diagram to show the definitions of the polar angle *θ* and azimuthal angle *φ* of single dipole generated on the AuNU surface in 3D space. (B) Schematic diagram depicting three-perpendicular dipoles along the three axes. *E*_a_ denotes the scattering electric field of the nanorod along the main long axis.


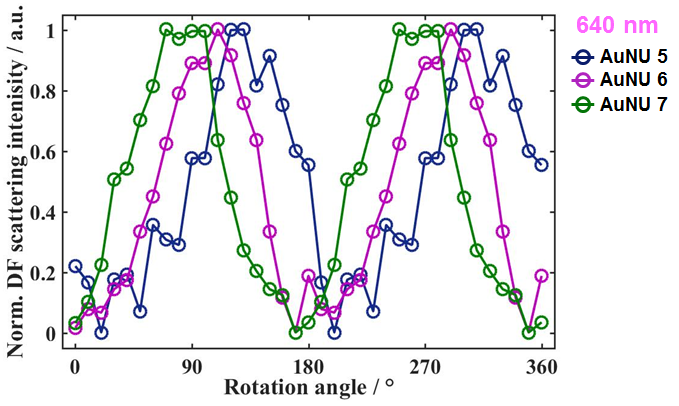


**Fig. S7** Normalized DF intensities for AuNUs (AuNU5 to AuNU7) at 640 nm as a function of the rotational angle


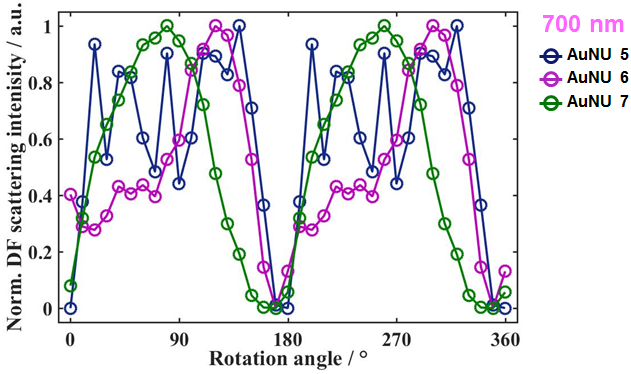


**Fig. S8** Normalized DF intensities for AuNUs (AuNU5 to AuNU7) at 700 nm as a function of the rotational angle
